# Negative Regulation of Violacein Biosynthesis in *Chromobacterium violaceum*

**DOI:** 10.3389/fmicb.2017.00349

**Published:** 2017-03-07

**Authors:** Giulia Devescovi, Milan Kojic, Sonia Covaceuszach, Miguel Cámara, Paul Williams, Iris Bertani, Sujatha Subramoni, Vittorio Venturi

**Affiliations:** ^1^International Centre for Genetic Engineering and BiotechnologyTrieste, Italy; ^2^Laboratory for Molecular Microbiology, Institute of Molecular Genetics and Genetic Engineering, University of BelgradeBelgrade, Serbia; ^3^Consiglio Nazionale delle Ricerche, Istituto di Cristallografia, U.O.S di TriesteTrieste, Italy; ^4^Centre for Biomolecular Sciences, School of Life Sciences, University of NottinghamNottingham, UK

**Keywords:** *Chromobacterium violaceum*, VioS, CviI/R quorum sensing, regulation, violacein, chitinase activity, protease activity

## Abstract

In *Chromobacteium violaceum*, the purple pigment violacein is under positive regulation by the *N*-acylhomoserine lactone CviI/R quorum sensing system and negative regulation by an uncharacterized putative repressor. In this study we report that the biosynthesis of violacein is negatively controlled by a novel repressor protein, VioS. The violacein operon is regulated negatively by VioS and positively by the CviI/R system in both *C. violaceum* and in a heterologous *Escherichia coli* genetic background. VioS does not regulate the CviI/R system and apart from violacein, VioS, and quorum sensing regulate other phenotypes antagonistically. Quorum sensing regulated phenotypes in *C. violaceum* are therefore further regulated providing an additional level of control.

## Introduction

Many Gram-negative bacteria regulate cell density dependent behavior by producing and sensing *N*-acylhomoserine lactone (AHL) signal molecules by a process called quorum sensing (QS; Fuqua et al., 1994). A canonical AHL-dependent QS system is composed of two proteins respectively belonging to the LuxI and LuxR protein families (Fuqua et al., 1996). Typically, AHLs are produced by an AHL synthase (LuxI homolog) and sensed at a threshold concentration due to increase in cell population density by an AHL-binding regulator (LuxR homolog) which then affects transcription of target genes (Fuqua and Greenberg, 2002). AHL QS regulates many phenotypes that impact on bacterial community or group behaviors including the expression of secreted enzymes, antibiotic and exopolysaccharide production, biofilm formation, conjugation, symbiosis, and virulence (Fuqua and Greenberg, 2002; Loh et al., 2002; Von Bodman et al., 2003; Waters and Bassler, 2005).

*Chromobacterium violaceum* is a betaproteobacterium found in a variety of soil and aquatic habitats causing infrequent but fatal mammalian infections (Brazilian National Genome Project, 2003). Two *C. violaceum* strains (ATCC31532 and ATCC12472) possess an AHL QS system and surprisingly they produce and respond to different AHLs (McClean et al., 1997; Morohoshi et al., 2008). The AHL QS system of *C. violaceum* ATCC12472 is encoded by the genetically linked *cviI* and *cviR* genes producing and responding with highest affinity to *N*-decanoyl-L-homoserine lactone (C10-HSL). CviR therefore binds to C10-HSL with highest affinity (Morohoshi et al., 2008; Swem et al., 2009) and the *cviI* AHL synthase is under positive feedback regulation by C10-HSL-CviR (Stauff and Bassler, 2011). The CviI/CviR QS system of *C. violaceum* ATCC12472 is important for virulence as revealed by loss of pathogenicity in a *C. elegans* infection model in the presence of an antagonistic ligand for CviR instead of C10-HSL (Swem et al., 2009). In contrast, a much earlier report (McClean et al., 1997) demonstrated that the AHL signal produced by *C. violaceum* ATCC31532 is C6-HSL. However, cloning and genetic analysis of this *cviI*/*cviR* QS system has not been yet been reported in detail.

In *C. violaceum*, QS regulates (i) the *vioA* promoter of violacein *vioABCDE* genes coding for the water insoluble purple pigment violacein (Lichstein and Van De Sand, 1946; McClean et al., 1997), (ii) genes coding for cyanide production and degradation (Durán and Menck, 2001), and (iii) multiple genes the products of which are chitinases (Chernin et al., 1998). Besides the *cviI* promoter, several other genes are directly regulated by CviR in *C. violaceum* ATCC12472 and these include genes coding for a putative transcriptional regulator (CV_0577), a guanine deaminase (CV_0578), a chitinase (CV_4240), and a type VI secretion system gene (CV_1432) (Stauff and Bassler, 2011). As in *C. violaceum* AHL QS regulates the production of the purple pigment violacein; this has allowed the convenient use of this bacterium as an AHL biosensor since the AHL-negative biosensor strain CV026 produces violacein only upon the addition of exogenous AHLs with from C4 to C8 acyl side chains (McClean et al., 1997; Steindler and Venturi, 2007).

Regulation of violacein production by QS has been studied in more detail than the other phenotypes as it is an easily discernible and visible trait. Using a combination of mutagenesis-based analysis in *C. violaceum* ATCC31532 and experiments in a heterologous *Escherichia coli* host, the *vioA* promoter of *vioABCDE* operon has been shown to be under the direct positive regulation of CviR (McClean et al., 1997; Swem et al., 2009). Comprehensive mutational analysis of the *vioA* promoter has also enabled the identification of a CviR binding site (Stauff and Bassler, 2011). Interestingly, the level of violacein produced by wild type *C. violaceum* ATCC12472 is much higher than that of wild type *C. violaceum* ATCC31532 (McClean et al., 1997). Furthermore, a violacein repressor has been reported and inactivated by transposon mutagenesis in two independent studies in *C. violaceum* ATCC31532 giving rise to mutants with considerably higher violacein production (McClean et al., 1997; Swem et al., 2009). In addition, the *Chromobacterium* AHL biosensor strain CV026 is a double transposon insertion mutant since single Tn5 insertions in the putative AHL synthase failed to respond to exogenous AHLs unless a second transposon was introduced into the putative repressor locus (McClean et al., 1997). However, the mechanism of violacein regulation by this putative repressor and its regulatory relationship with the *C. violaceum* AHL QS system are not known.

In this study we have examined the regulation of violacein production in *C. violaceum* ATCC31532 and characterized its QS system as well as a repressor mutant of this strain with respect to violacein production. We show that the expression of the *vioA* promoter of the *vioABCDE* operon is under negative regulation by this novel repressor which we have named VioS. VioS is also involved in the regulation of other AHL QS regulated phenotypes such protease and chitinolytic activity. Furthermore, we provide evidence for direct interference by VioS of QS mediated positive regulation of the *vioA* promoter in *C. violaceum* and in *E. coli*. Finally, we show that VioS functions as a repressor of violacein production in the closely related *C. violaceum* ATCC12472 when introduced *in trans*. We propose that VioS is a novel protein that functions to fine-tune the QS regulated phenotype of violacein biosynthesis by regulating *vioA* promoter expression rather than modulating the regulation of *cviI/cviR* gene expression.

## Materials and methods

### Bacterial strains, media, and growth conditions

Wild type *C. violaceum* ATCC 31532, ATCC12472, and CV026 (McClean et al., 1997) and *Escherichia coli* strains DH5α and M15 were routinely grown at 30°C and 37°C, respectively, in Luria–Bertani (LB) broth medium (Miller, 1972). When required, antibiotics were added in the following concentrations: ampicillin 100 μg ml^−1^, kanamycin 100 μg ml^−1^, gentamicin 50 μg ml^−1^, tetracyclin 40 μg ml^−1^ for *C. violaceum* strains and, ampicillin 100 μg ml^−1^, kanamycin 50 μg ml^−1^, gentamycin 20 μg ml^−1^ and tetracyclin 20 μg ml^−1^ for *Escherichia coli* strains. AHLs used here were obtained from Sigma-Aldrich (St. Louis, MO, USA).

### Recombinant DNA techniques

DNA manipulations, including digestion with restriction enzymes, agarose gel electrophoresis, purification of DNA fragments, ligation with T4 DNA ligase, transformation of *E coli*, colony hybridization and radioactive labeling by random priming, were performed as previously described (Sambrook et al., 1989). Plasmids were purified using EuroClone columns (EuroClone S.p.A., Italy). Total DNA from *C. violaceum* was isolated with the sarkosyl-pronase lysis method (Better et al., 1983). Triparental matings to mobilize DNA from *E. coli* to *C. violaceum* were carried out with the helper strain *E. coli* (pRK2013) (Figurski and Helinski, 1979). PCR amplifications were performed on *C. violaceum* ATCC31532 genomic DNA using GoTaq Flexi DNA Polymerase (Promega, Madison, WI, USA).

### Plasmid construction

The plasmids used in this study are listed in Table 1.

**Table 1:** **Strains, plasmids, and primers used**.

**Strains/plasmids/primer**	**Relevant features**	**References or sources**
***C. violaceum* STRAINS**		
*C. violaceum* ATCC31532	WT isolate	
*C. violaceum* ATCC12472	WT isolate	
CV026	Double transposon mutant of ATCC31532, violacein and AHL negative	McClean et al., 1997
MB8	*vioS*::Tn*5* of *C. violaceum* ATCC31532; Km^R^	This study
MB11	*vioS*::Tn*5* of *C. violaceum* ATCC31532; Km^R^	This study
31532VIOS	*vioS*::Km of *C. violaceum* ATCC31532; Km^R^	This study
31532CVII	*cviI*::Km of *C. violaceum* ATCC31532; Km^R^	This study
31532CVIR	*cviR*::Gm of *C. violaceum* ATCC31532; Gm^R^	This study
**PLASMIDS**		
pRK2013	Tra^+^ Mob+ColE1 replicon; Km^R^	Figurski and Helinski, 1979
pGEM2T	Cloning vector; Amp^R^	Promega
pMP220	Promoter probe vector, IncP; Tc^R^	Spaink et al., 1987
pQE30	Expression vector; Amp^R^	Qiagen
pBSIIKS	Cloning vector; Amp^R^	Stratagene
pBBRmcs5	Broad-host-range vector; Gm^R^	Kovach et al., 1995
pKNOCK-Km	Conjugative suicide vector; Km^R^	Alexeyev, 1999
pKNOCK-Gm	Conjugative suicide vector; Gm^R^	Alexeyev, 1999
pSUP2021	Tn*5* delivery suicide plasmid; ColE1; Km^R^	Simon et al., 1983
pLAFR3	Broad-host-range vector, IncP; Tc^R^	Staskawicz et al., 1987
pCVO7	pLAFR3 containing *C. violaceum* 31532 DNA; Tc^R^	This study
pBSCVO7H	pBSIIKS carrying a HindIII 3 kb fragment from CVO7; Amp^R^	This study
pBCVO7XN	pBSIIKS carrying a XhoI-NotI 6.35 kb fragment from CVO7; Amp^R^	This study
pBBVioS	pBBRmcs5 containing VioS ; Gm^R^	This study
pKNOCKcviI	Internal *cviI* fragment cloned in pKNOCK-Km	This study
pKNOCKcviR	Internal *cviR* fragment cloned in pKNOCK-Gm	This study
pKNOCKvioS	Internal *vioS* fragment cloned in pKNOCK-Km	This study
pMPGFP	pMP220 containing the GFPmut3 gene deprived of its promoter	This study
pPcviIGFP	*cviI* promoter cloned in pMPGFP	This study
pPcviRGFP	*cviR* promoter cloned in pMPGFP	This study
pPvioAGFP	*vioA* promoter cloned in pMPGFP	This study
pPvioSGFP	*vioS* promoter cloned in pMPGFP	This study
pBBRcviR	*cviR* cloned in pBBRmcs5	This study
pQE30VioS	*vioS* cloned in pQE30	This study
pPvioA220	*vioA* promoter cloned in pMP220	This study
pPcepI220	*cepI* promoter cloned in pMP220	This study
pscR2	pQF50 vector expressing the B. cepacia cepR gene	Aguilar et al., 2003
pMP77	Promoter probe vector; IncQ; CmR	Spaink et al., 1987
pMPCviRLacZ	*cviR* translational fusion	This study
pMPVioALacZ	*vioA* translational fusion	This study
**PRIMERS**		
Primers name	Sequence	Source
cviIBF	GGATCCCCGTAGGCAAAGAACTAA	This study
cviIER	GAATTCTTGTGTCTGAACGCCA	This study
cviRBF	GGATCCCCGAAACTCATCCAAAAA	This study
cviRER	GAATTCGTTGATGGGTTTCGAGAT	This study
vioABF	CGGATCCGTGTTGCATTTCTCAAATGG	This study
vioAER	GGAATTCGAAGAGTGCTTCATCACGA	This study
vioSBF	GGATCCGCCCAAAGCCAGACTA	This study
vioSER	GAATTCTGAACGGCACGATTGA	This study
GFPEF	GGAATTCAAGAGGAGAAATTAAGATG	This study
GFPPR	ACTGCAGTCAGCTAATTAAGCTTATT	This study
vioA220KF	AGGTACCGTGTTGCATTTCTC	This study
vioA220XR	GTCTAGAGAAGAGTGCTTCAT	This study
cepI220EF	GAATTCTCGCTTACGTGACGGTCG	This study
cepI220XR	TCTAGAGCATGGTGTCCTCGGATT	This study
cviRPROMF_Xba	TCTAGAGCCGAAACTCATCCAAAA	This study
cviR2R_BglII	AGATCTGGGCGTAGTTTTCCTCATGT	This study
vioAPROMF_Xba	GTCTAGAAAATGGAAAGCCTGTCACT	This study
vioAR3_BamHI	AGGATCCTCTGCATGTCGAAAAT	This study
VioSBFw	AGGATCCCCTTGCATCACCCGCAGT	This study
VioSHR	GAAGCTTTTACGAGGCGGGGTTTAGA	This study
cviRHF	CAAGCTTCAAGGAAGACTCGCTCAT	This study
cviRXR	GTCTAGATCATTCGTTCGCTACGGT	This study
KncviIBF	AGGATCCAGGCTATTGGTGCC	This study
KNcviIKR	AGGTACCAGCCGGCGGTACAT	This study
KNcviRF	CCAGAACCAGATCCAGCG	This study
KNcviRR	GATGGACAGGATGCTGCCG	This study
KNvioSKF	AGGTACCCGGCTGCACGAAGC	This study
KNvioSBR	AGGATCCCAGGCAAGCCAGC	This study

The *gfp* reporter gene was chosen for studying the promoter activities in *C. violaceum* in order to reduce to the minimum, possible, interference by violacein that can be an issue with the β-galactosidase assay. A *gfp* based reporter plasmid was constructed by amplifying the *gfp* gene, deprived of its promoter, from plasmid pBBR2-GFP (Passos da Silva et al., 2014) using the primers GFPEF and GFPPR. The amplified *gfp* was then cloned as an *Eco*RI/*Pst*I fragment in pMP220 vector, generating pMPGFP.

Gene transcriptional fusion plasmids, based on the pMPGFP promoter probe vector, were constructed as follows: the promoter regions of *cviI, cviR, vioA*, and *vioS* genes were amplified from *C. violaceum* 31532 genomic DNA by using, respectively, the primers cviIBF and cviIER (cviIPROM; 337-bp), cviRBF, and cviRER (cviRPROM; 277-bp), vioABF and vioAER (vioAPROM; 328-bp), vioSBF, and vioSER (vioSPROM; 196-bp). The amplified fragments were cloned in pGEM-T Easy vector (Promega, Madison, WI, USA), sequenced and then excised as *Bam*HI/*Eco*RI) fragments and cloned into the *Bgl*II/*Eco*RI sites in pMPGFP obtaining pPcviIGFP pPcviRGFP, pPvioAGFP, and pPvioSGFP constructs. The *vioA* promoter was also amplified with primers vioA220KF and vioA220XR and cloned as a *Kpn*I/*Xba*I fragment into the corresponding restriction sites of the promoter probe vector pMP220, obtaining pPvioA220 construct. The *cepI* promoter was amplified with primers cepI220EF and cepI220XR and cloned as a *Eco*RI/*Xba*I fragment in pMP220 giving pPcepI220. The *vioS* gene with its promoter was cut out from the pBSCVO7H construct as a *Sna*BI/*Xba*I fragment and cloned into the corresponding restriction sites of pBBRmcs5 to generate pBBRvioS. The *vioS* gene was also amplified from *C. violaceum* 31532 genomic DNA using the primers VioSBFw and VioSHR and cloned into the *Bam*HI/*Hin*dIII restriction sites of pQE30 vector to generate pQE30VioS. The *cviR* gene was amplified from *C. violaceum* 31532 genomic DNA using primers cvRHF and cvRXR and inserted downstream of the *lac* promoter in pBBRmcs5 linearized with *Hin*dIII and *Xba*I restriction enzymes. The fidelity of all of the constructs described was verified by DNA sequencing (Macrogen, Europe).

Translational fusions were constructed as follows: the 5′ region of the *cviR* DNA sequence, containing the promoter and coding sequences for the first 98 amino acids was amplified from *C violaceum* 31532 genomic DNA by using the primers cviRPROMFXba and cviR2RBglII. The amplified fragment was then cloned in frame upstream from the *lacZ* gene and then the whole construct was transferred into the pMP77 vector generating pMPCviRLacZ. Similarly, the 5′ region of the *vioA* gene, containing the promoter and coding sequences for the first 49 amino acids was amplified by using the primers vioAPROMFXba and vioAR3BamHI, cloned in-frame upstream the *lacZ* gene and transferred to the pMP77 plasmid giving pMPVioAlacZ.

### Genomic mutant bank and cosmid gene bank construction and screening

A Tn*5* genomic mutant library of *C*. *violaceum* ATCC31532 was created using pSUP2021, as previously described (Simon et al., 1983). Approximately 5,000 mutants were screened for the presence of violacein hyperproducer mutants by identifying colonies that showed purple coloration in contrast to the pale colonies of the *C*. *violaceum* ATCC31532 wild type. Two mutants were isolated and the genomic regions flanking the Tn*5* insertions were amplified by arbitrary PCR technique (O'Toole and Kolter, 1998) and sequenced. The two mutants were designated as MB8 and MB11 respectively. A genomic bank (cosmid library) of *C. violaceum* ATCC31532 was constructed as follows. Briefly, *C. violaceum* 31532 genomic DNA was partially digested with *Eco*RI and ligated into pLAFR3 cosmid vector. The constructs obtained were introduced into *E. coli* cells using Gigapack III XL-4 packaging kit as recommended by the supplier (Stratagene-Agilent, Santa Clara, CA, USA). The genomic bank was then screened using the flanking DNA (obtained by arbitrary PCR on mutant colonies MB8 and MB11), as probes. Three cosmids were isolated which showed the same restriction pattern. Cosmid pCVO7 was chosen and subcloned in pBSIIKS generating two overlapping constructs: pBCVO7H (containing a 3-kb *Hin*dIII fragment) and pBCVO7XN (containing a 6,350-bp *Xho*I-*Not*I fragment).

### Construction of 31532CVII, 31532CVIR, and 31532VIOS

The three additional mutants, 31532CVII, 31532VCIR, and 31532VIOS were generated using the suicide vectors from the pKNOCK series (Alexeyev, 1999). To generate 31532cviI, an internal fragment (209-bp) of the *cviI* gene was PCR amplified using the primers KNcviIBF and KNcviIKR and cloned as a *Bam*HI-*Kpn*I fragment into the corresponding sites of pKNOCK-Km resulting in pKNOCKcviI. In order to generate 31532CVIR, an internal fragment of *cviR* (327-bp) was amplified with the primers KNcviRF and KNcviRR, blunted and cloned into pKNOCK Gm digested with the *Sma*I restriction enzyme, yielding pKNOCKcviR. Finally, to obtain 31532VIOS, an internal fragment of *vioS* (187-bp) was amplified with primers KNvioSKF and KNvioSBR and cloned as a *Kpn*I-*Bam*HI fragment in the corresponding sites of pKNOCK-Km giving pKNOCKvioS. The pKNOCK constructs obtained were transferred to *C. violaceum* ATCC31532 via tri-parental mating and the knock-out mutants were verified by PCR analysis and sequencing. The 31532VIOS was altered in growth rate and behaved like the parent wild-type strain.

### Extraction and quantification of AHLs

*C. violaceum* strains were grown overnight in 20 ml of LB medium. The cells were pelleted at 5,000 *g* for 15 min. The cell free supernatants were filtered (using 0.45 μm filters; Millipore) and extracted twice with an equal volume of ethyl acetate containing 0.1% v/v acetic acid. The organic phases were collected, dried to completeness and re-suspended in 50 μl of ethyl acetate. To quantify the amounts of C6-HSL produced by the 31532 wild type strain, MB8, MB11, and 31532VIOS, the constructs pPvioA220 and pBBRcviR were used to constitute a CviR-based sensor regulating its target promoter *vioA* in the heterologous *E. coli* M15 system. In order to generate a calibration curve, different concentrations (0; 0.01; 0.05; 0.1; 0.5; 1 μM) of C6-HSL were added to 10 ml to each of the sensor strains. The cultures were grown for 6 h and β-galactosidase activity was determined. To quantify the AHLs produced by each *C. violaceum* strain, the experiment was repeated by adding 10 μl of an AHL extract obtained from each strain to the sensor.

### β-galactosidase and GFP quantification assays

β–galactosidase activities were determined essentially as described by Miller (1972), with the modifications of Stachel (Stachel et al., 1985). Each experiment was performed in triplicate. GFP fluorescence in the stationary phase of the bacterial cultures was determined in a Perkin Elmer EnVision Multilabel Reader that was set to an excitation wavelength of 485 nm and an emission wavelength of 510 nm.

### Exoenzyme activity

To assess protease activity, *C. violaceum* strains were grown to stationary phase and 2 μl of culture was spotted onto M9 agar containing 2% dry milk, as the only carbon source. Zones of activity were measured after 36 h. For chitinase activity, the same protocol was followed and cultures were spotted onto M9 agar containing 0.2% colloidal chitin (Ahmadian et al., 2007).

## Results

### The AHL QS system of *C. violaceum* ATCC31532

The unequivocal chemical identification of C6-HSL from culture supernatants of *C. violaceum* ATCC 31532 and the selection of a Tn5 transposon mutant with an insertion in a putative *luxI* orthologue demonstrated the presence of an AHL QS system in this organism (McClean et al., 1997). To isolate the locus encoding this system, a *Pst*I genomic library of this strain was constructed in pUC18. The library was introduced into the AHL biosensor strain *E. coli* (pSB401) (Winson et al., 1998) and the recombinant colonies screened for the production of bioluminescence using a photon-imaging camera as previously described (Swift et al., 1997). A recombinant clone (pMW50) able to induce light production in the biosensor strain was identified as a highly bioluminescent colony. Expression of pMW50 in *E. coli*, was able to restore violacein production when cross-streaked against the AHL sensor strain *C. violaceum* CV026 (McClean et al., 1997) suggesting the presence of an AHL synthase in this recombinant clone. Sequence analysis of the 6 Kb *Pst*I insert from pMW50 revealed the presence of two convergent open reading frames overlapping by 74 bp which were named *cviR* and *cviI* as their predicted amino acid sequences were homologous to the LuxI/LuxR family of QS genes. Solvent extraction of culture supernatants from *E. coli* harboring pMW50 followed by LC-MS/MS analysis revealed the presence of C6-HSL (data not shown). No other AHLs were detected from these extracts indicating that *cviI* is responsible for the synthesis of this AHL.

### Violacein biosynthesis is negatively regulated by VioS

**Figure 1:**
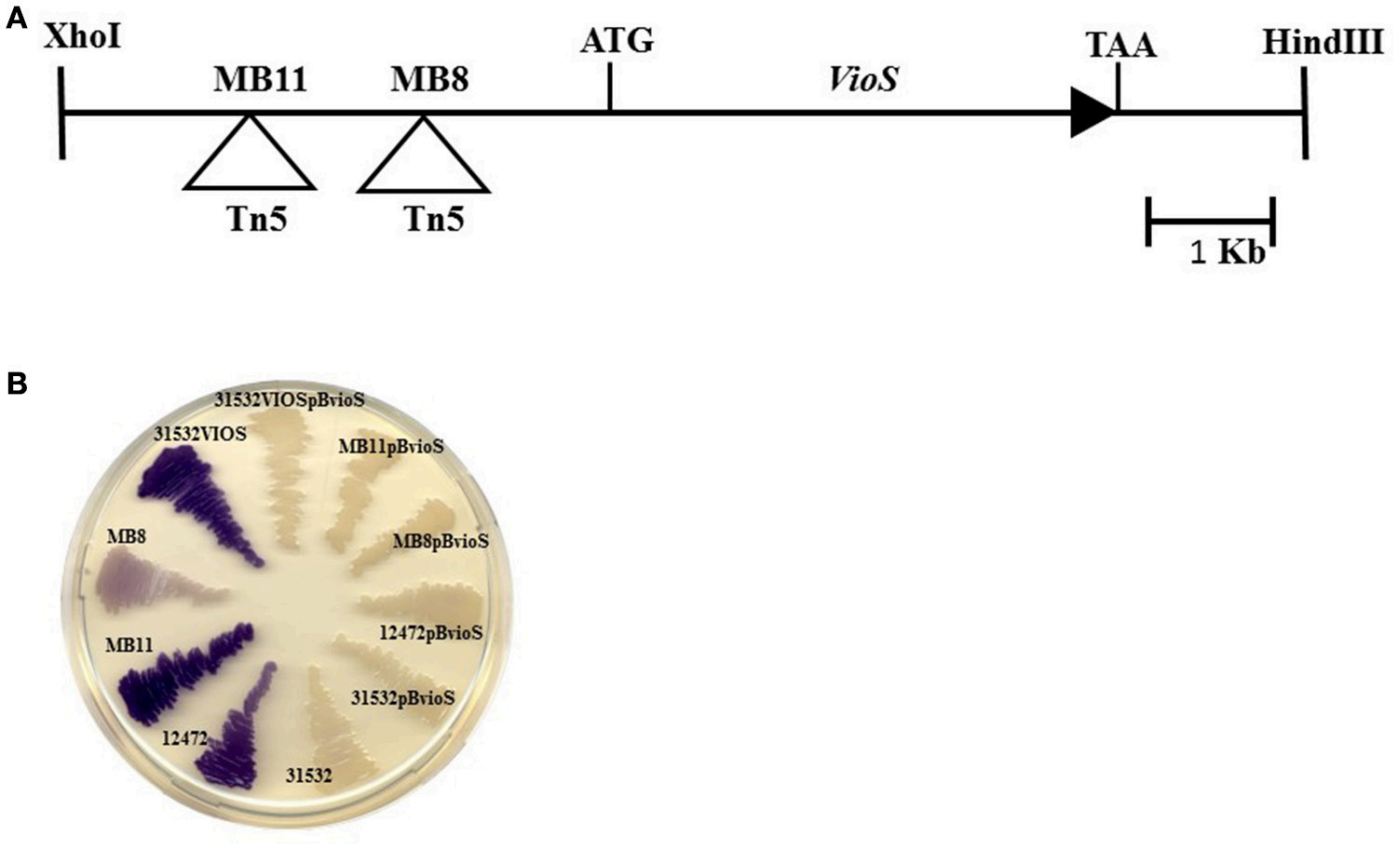
**(A)** Genomic organization of the *vioS* locus. The Tn*5* insertions in mutants MB8 and MB11 are indicated. **(B)** Production of violacein in *C. violaceum* wt strains ATCC31532 and ATCC12472, 31532VIOS mutants (MB8; MB11;31532VIOS), and mutants complemented with pBBVioS, containing full length *vioS*.

Violacein production by *C. violaceum* is regulated by QS via AHLs signal molecules (McClean et al., 1997; Morohoshi et al., 2008). We have previously shown that violacein production is stringently negatively regulated since we obtained a Tn5 insertion mutant that strongly overproduced violacein in the *C. violaceum* ATCC31532 genetic background (McClean et al., 1997; Table 1). This transposon was localized to a gene coding for a protein of unknown function homologous to CV_1055 of the sequenced genome of *C. violaceum* ATCC12472 demonstrating that violacein is very tightly regulated (Swem et al., 2009). To further investigate the regulation of this phenotype and to make sure that no other loci was involved in this negative regulation, we constructed a Tn*5* mutant library of *C*. *violaceum* ATCC31532 and screened for more mutants that overproduced violacein as described in the Materials and Methods. Two mutants, named MB8 and MB11 were identified in the screen and the location of the Tn*5* insertion site in both mutants was also located in the CV_1055 gene homolog from *C. violaceum* ATCC12472 but in the putative promoter region; the Tn5 in mutant MB8 is located nearer to the ATG of the putative ORF whereas MB11 is further away (Figure 1A). We have now named the hypothetical protein encoded by this gene as VioS (Figure 1A). This predicted protein (138 amino acids; 15 kDa approximately) showed 91% identity and 94% similarity to a hypothetical protein from *Pseudogulbenkiana ferrooxidans* and 85% identity and 90% similarity to the hypothetical protein encoded by CV_1055 from *C. violaceum* ATCC12472 respectively. Conserved domain analysis of VioS amino acid sequence revealed the presence of a domain of unknown function annotated as DUF1484 spanning 32–138 amino acids (8.35e-03) that is exclusively found in bacteria belonging to the betaproteobacteria.

Both MB8 and MB11 transposon mutants exhibited increased violacein production in contrast to the pale white color of *C. violaceum* ATCC31532 wild type (Figure 1B). Mutant MB11 displayed a much stronger violet color compared with MB8 indicating that the transposon insertion in MB11 resulted in greater violacein production. As neither transposon insertion was located in the putative structural gene, an insertion mutant in the putative *vioS* ORF was generated (designated as 31532VIOS) as described in the Materials and Methods. This mutant showed violacein overproduction similar to MB8 (Figure 1B). Complementation of mutants MB8, MB11, and 31532VIOS with a plasmid construct containing full length *vioS* and flanking upstream DNA restored violacein production in all the mutants to wild type levels (Figure 1B). These results strongly suggest a role for VioS in the negative regulation of violacein biosynthesis in *C. violaceum* ATCC31532.

### VioS and CviR regulate violacein biosynthesis in opposite ways

**Figure 2:**
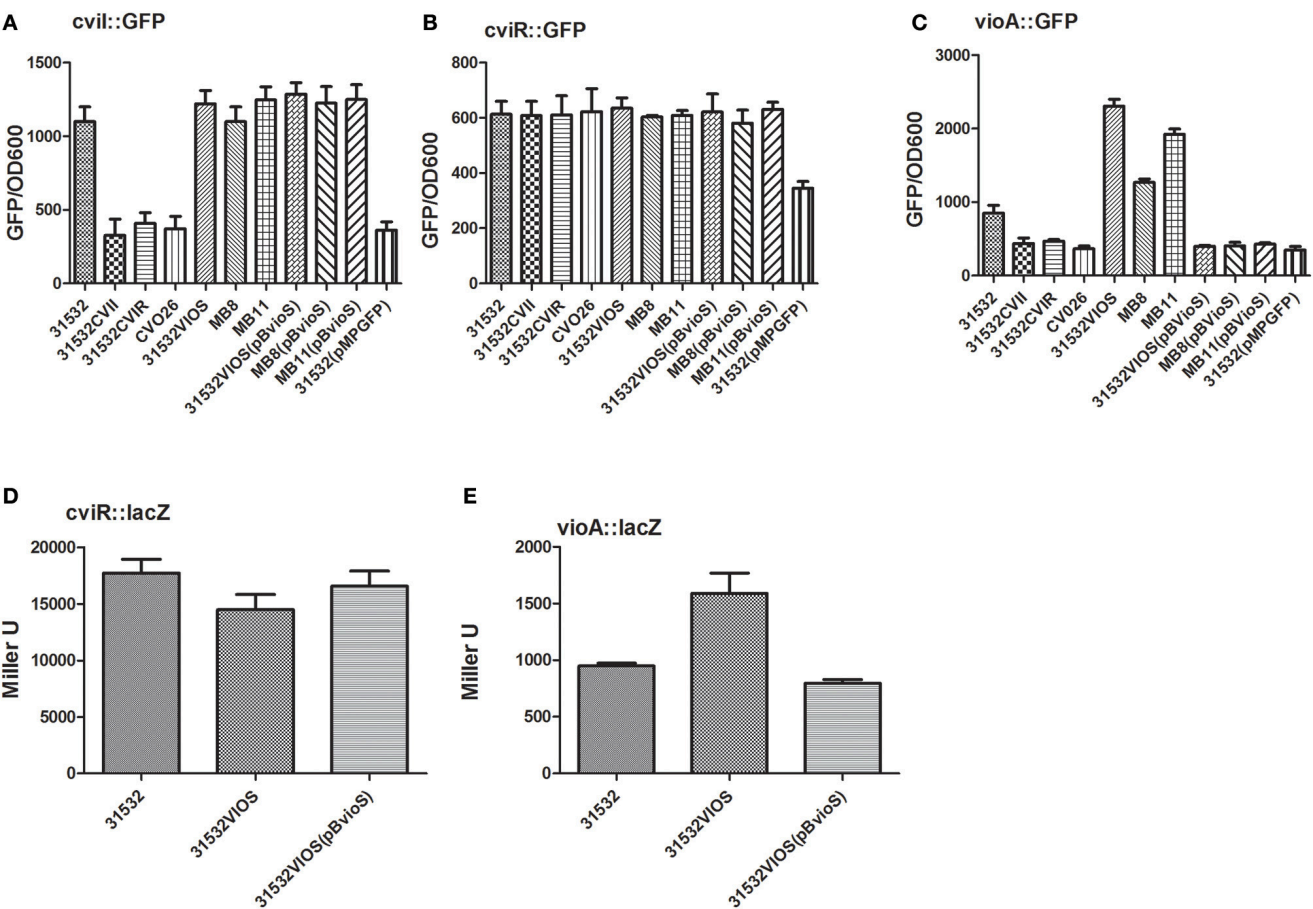
**CviI/R QS system is not influenced by VioS but VioS negatively regulates the expression of the *vioA* operon**. CviI promoter activity **(A)**, cviR promoter activity **(B)**, and *vioA* promoter activity **(C)** in *C violaceum* 31532, 31532 quorum sensing mutants, 31532VIOS mutants (MB8; MB11; 31532VIOS) and mutants complemented with pBBVioS, containing full length *vioS*. Stationary phase bacterial cultures were monitored for GFP expression in a Perkin Elmer EnVision Multilabel Reader. The means plus standard deviations for five replicates are shown and 31532 (pMPGFP) represents the empty vecotr. In panels **(D,E)**, β-galactosidase levels for the *cviR* and *vioA lacZ* translational fusions are shown in *C. violaceum* 31532 wild-type, in the *vioS* mutant 31532VIOS and in the same mutant complemented with a plasmid—borne copy of *vioS* gene.

Since the studies using the transposon insertion mutants described above clearly support a role for VioS in the negative regulation of violacein production, which conversely is positively regulated by the CviI/R QS system, we sought to determine whether VioS interacted with the QS system. Consequently we investigated whether VioS influenced the expression of the CviI/R system which could then result in violacein de-regulation. We first determined the AHL levels produced by the wild type, MB8, MB11, and 31532VIOS strains as described in the Materials and Methods. Using a calibration curve derived by a CviI/R AHL biosensor constructed here, we found that all strains produced similar AHL levels production corresponding to a C6-HSL concentration of approximately 0.5 μM (data not shown). The transcriptional levels of the QS genes using *cviI*::*gfp* and *cviR*::*gfp* plasmid transcriptional fusions were determined and the results showed that the *cviI* and *cviR* genes are expressed at comparable levels in the wild type, the *vioS* mutants and complemented strains (Figures 2A,B). To determine whether the CviI/R QS system modulated *vioS* expression, assays were carried out to measure the levels of a plasmid-borne *vioS*::*gfp* transcriptional fusion in the wild type, *cviI* and *cviR* mutants. The expression of *vioS* was similar in all of the strains examined (data not shown). These results indicate that VioS does not influence expression of the CviI/R QS system or vice versa. VioS furthermore does not significantly affect the levels of AHLs.

To further understand the opposing regulatory effects of VioS and CviR-AHL on violacein production we monitored the reporter activity of a plasmid *vioA*::*gfp* transcriptional fusion in the wild type, MB8, MB11, 31532VIOS, *cviI*, and *cviR* mutants (Figure 2C). The *vioA* promoter controls the expression of the operon (*vioA-vioE*) encoding for the violacein biosynthesis genes (August et al., 2000; Antônio and Creczynski-Pasa, 2004; Sánchez et al., 2006). As expected, little expression of *vioA*::*gfp* was apparent in the *cviI* and *cviR* mutants compared with the wild type. On the other hand *vioA*::*gfp* fusion showed a drastic increase in expression in all three *vioS* mutants, MB8, MB11, and 31532VIOS compared with the wild type strain. Complementation of the *vioS* mutants with a wild type copy of the *vioS* gene restored *vioA*::*gfp* expression to wild type levels (Figure 2C). These results demonstrate that VioS represses expression of the *vio* operon at the transcriptional level thus influencing violacein production in the *C. violaceum* ATCC31532 wild type strain in spite of presence a functional CviI/R QS system.

To investigate whether VioS has an effect on the translational levels of *cviI* and *vioA*, we constructed *cviR-lacZ* and *vioA-lacZ* translational fusions as described in the Materials and Methods. As depicted in Figures 2D,E, VioS did not affect *cviR* translation. However, in the *vioS* mutant, the *vioA-lacZ* translational fusion displayed a 2-fold increase in β-galactosidase activity. These data indicate that VioS exerts a negative effect on the translation of *vioA* meaning that it could be acting at a post-transcriptional level; however this increase in translation could be due to the increase in transcription observed using the *vioA* transcriptional fusion (Figure 2C).

### VioS is sufficient to antagonize CviR-mediated regulation of the violacein biosynthetic operon in a heterologous system

**Figure 3:**
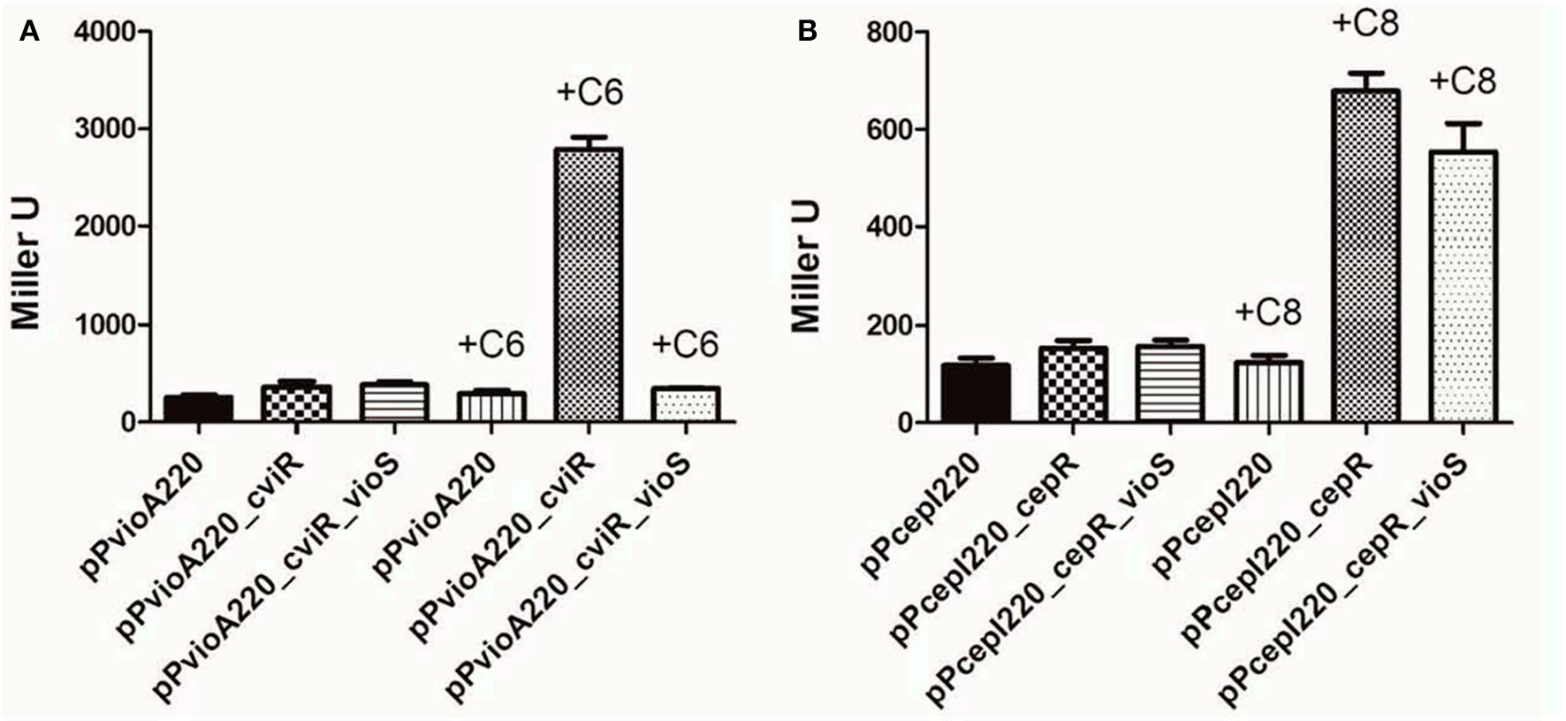
**VioS antagonizes CviR mediated activation of the *vioA* promoter in a heterologous *E. coli* strain and this inhibition is specific. (A)** *vioA* promoter activity in the presence of CviR alone or together with VioS, in the absence/presence of C6-HSL (1 μM). **(B)** *cepI* promoter activity in the presence of CepR alone or together with VioS, in the absence/presence of C8-HSL (1 μM). β-gal activities were measured after 12 h of growth. Experiments were performed in triplicate and means plus standard deviations are plotted.

To determine whether VioS is sufficient to antagonize CviR-mediated positive regulation of the *vio* operon, the entire system consisting of VioS, CviR, and the target promoter *vioA*::*lacZ* was reconstructed and introduced into a heterologous *E. coli* strain as described in Materials and Methods (Figure 3A). When the activity of *vioA*::*lacZ* fusion was monitored in *E. coli* in the presence of CviR and C6-HSL, the promoter showed high levels of expression consistent with CviR the positively regulating *vioA* in the presence of the cognate AHL signal.

The increased *vioA*::*lacZ* expression was not observed in the absence of C6-HSL. Upon expression of VioS in the same *E. coli* strain containing CviR and exogenously added C6-HSL, *vioA*::*lacZ* expression was reduced by over 6-fold indicating that VioS antagonizes the action of CviR, repressing *vioA* promoter activity. This observation in a heterologous system also indicates that VioS alone is sufficient to mediate the negative regulation of the *vioA* promoter.

It was also of interest to establish whether the negative effect of VioS on transcription of an AHL QS target gene was specific for the CviR regulated *vioA* promoter. Expression studies were therefore carried out using a different AHL QS system and target promoter. For this experiment we used the *Burkholderia cepacia* CepI/R system and the *cepI* target gene. The plasmid *cepI*::*lacZ* transcriptional fusion construct was introduced into *E. coli* harboring plasmids expressing either CepR or VioS. The expression of the *cepI::lacZ* fusion was determined with and without the exogenous addition of C8-HSL. In this experiment, the *cepI* promoter was upregulated in the presence of CepR and AHLs as expected but in contrast to the *vioA* promoter, it was not repressed in the presence of VioS (Figure 3B). Thus, the VioS mediated effect on the expression of a QS regulated promoter is likely to be specific for the CviI/R system.

### QS and VioS antagonistically modulate QS-regulated phenotypes in *C. violaceum*

**Figure 4:**
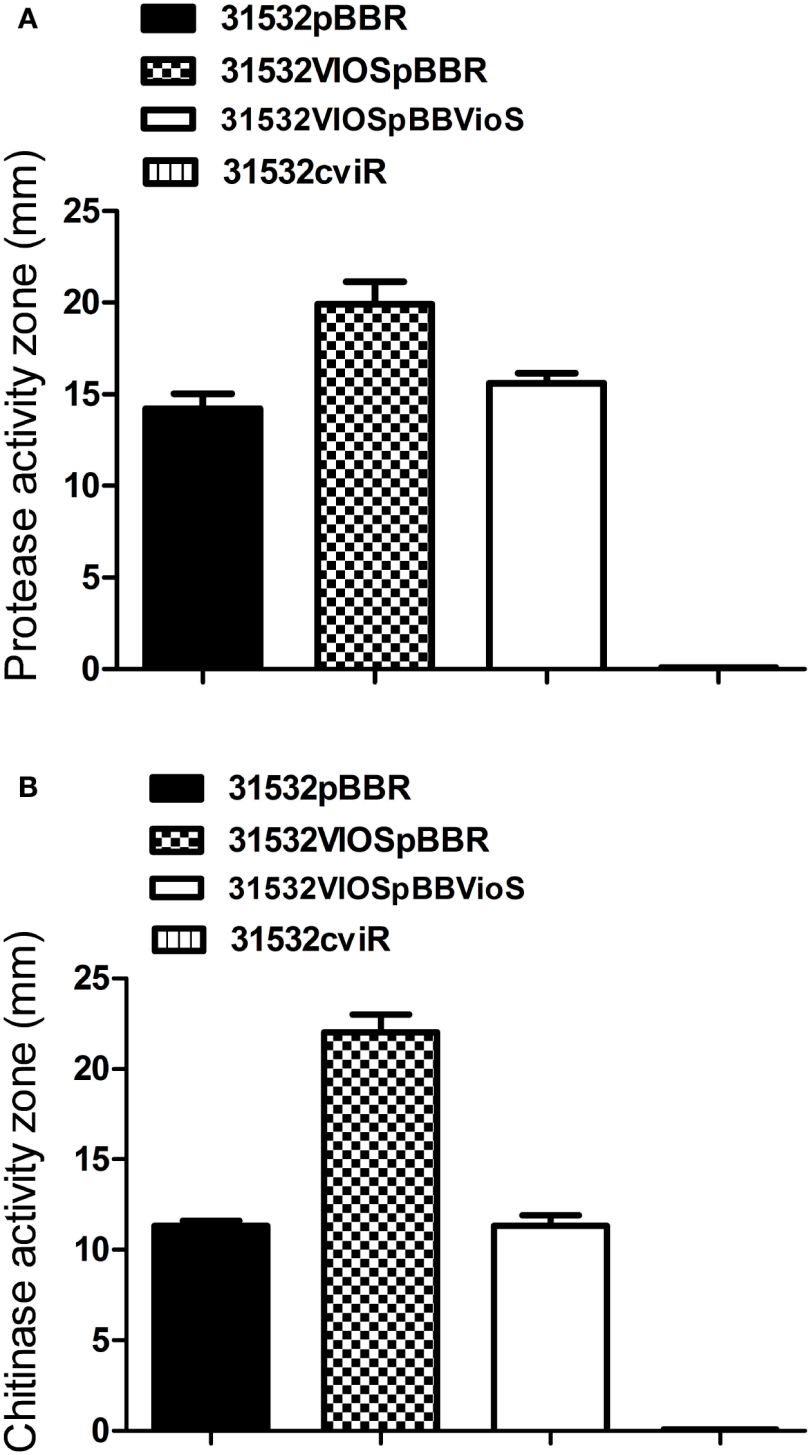
**Effect of *vioS* and *cviR* mutations on (A)** protease activity and **(B)** chitinase activity. The halos of cleared zones in milk agar plates were measured after 36 h growth. The halos of cleared zones in colloidal chitin plates were measured after 3 days of incubation. Experiments were performed in triplicate and means plus standard deviations are presented.

Since VioS negatively regulates violacein production, we investigated whether it plays a role in fine-tuning the expression of other QS-regulated phenotypes in *C. violaceum*. Protease and chitinolytic activities are known to be positively regulated by the CviI/R QS system in *C. violaceum* (Chernin et al., 1998). In the *cviR* mutant of ATCC31532 both protease and chitinase activities were abolished when compared with the wild type. In contrast to this, the *vioS* mutant showed increased levels of both protease and chitinase activities which could be reduced back to wild type levels by providing VioS *in trans* (Figures 4A,B). This shows that VioS also acts as a repressor of these two CviI/R QS regulated phenotypes as well as of violacein production. VioS might therefore play a more general role in adjusting the expression of CviI/R QS target genes in a manner opposite to their regulation by CviI/R QS.

## Discussion

In this study we report the regulatory functions of VioS, a putative repressor protein that negatively controls violacein production without influencing expression of the CviI/R QS system. The repressor function of VioS on violacein production is dominant as it antagonizes positive regulation by CviR/C6-HSL in wild type *C. violaceum* ATCC31532. Other phenotypes positively regulated by CviR-AHL, including protease and chitinase production, were also negatively regulated by VioS. Our results have thus uncovered a novel repressor of *C. violaceum* QS and identified another layer of population dependent regulation in this bacterium.

*C. violaceum* is an environmental bacterium, found in soil and water, is generally non-pathogenic but occasionally extremely virulent to humans and animals (Brazilian National Genome Project, 2003). It has been shown that elimination of QS leads to loss of virulence of *C. violaceum* in a *C. elegans* model of infection suggesting that functions positively regulated by QS are important for infection (Swem et al., 2009). However, the phenotypes regulated by AHL-dependent QS can be energetically expensive such that constitutive expression of these shared traits is not likely to enable optimal utilization of available resources; it may also elicit stronger host defense responses. RsaL, a negative regulator of QS and QS-regulated genes in *Pseudomonas aeruginosa* has been reported to be important for optimum virulence as *rsaL* mutants are hypervirulent in a *Galleria mellonella* acute model of infection (Rampioni et al., 2009). Also, in a study involving dual-species co-culture of *C. violaceum* and *Burkholderia thailandensis*, it was reported that QS dependent antimicrobials like violacein can provide a competitive advantage in mixed microbial communities with limited nutrients (Chandler et al., 2012). Here, we have shown that VioS functions to fine-tune QS-regulated phenotypes and it is possible that it might play a role in providing optimum fitness to *C. violaceum* both in the environment and in host associations. Alternatively, it cannot be excluded that VioS responds to environmental stimuli or an unknown signal that results in de-repression and so promotes high levels of violacein production under certain circumstances.

Although the molecular mechanism of VioS-mediated repression in QS homeostasis is not known, it is possible that it belongs to a new class of regulators. Among the few characterized negative regulators of QS are RsaL, AlgQ, and a TetR-like transcriptional repressor of *P. aeruginosa*, all of which bind DNA (De Kievit et al., 1999; Ledgham et al., 2003; Rampioni et al., 2006; Venturi et al., 2011; Longo et al., 2013). RsaM of *P. fuscovaginae* as well as other repressor proteins with less sequence identity to RsaM including BcRsaM of *B. cenocepacia* and TofM of *B. glumae* are also QS repressors (Mattiuzzo et al., 2011; Chen et al., 2012; Michalska et al., 2014). However, BcRsaM is predicted to influence QS by an as yet unknown mechanism but not by binding to DNA (Michalska et al., 2014). The VioS amino acid sequence does not show similarity to any of these proteins and furthermore this study does not provide any direct evidence that VioS exerts its regulation at the transcriptional level. Studies performed using translational fusions indicate that VioS had a negative effect on the translation of *vioA*. A comparison with RsaL of *P. aeruginosa* suggests that VioS exhibits some common and distinct features. The *rsaL* gene is genetically linked to QS systems and its transcription is positively regulated by QS. However, RsaL negatively regulates expression of *lasI* coding for AHL synthase as well as some other QS regulated genes responsible for e.g., pyocyanin and HCN production (Schuster et al., 2004; Rampioni et al., 2006, 2007b). RsaL and LasR have been shown to bind to adjacent sites on the *lasI* promoter but the negative regulatory effect of RsaL is dominant over the activating effect of LasR-AHL (Rampioni et al., 2007a). In our study, the presence of VioS influences *vioA* promoter activity in a manner similar to RsaL-mediated repression of the *lasI* promoter because the repressor activity of VioS on *vioA* promoter is dominant over the activator effect of CviR-AHL. However, unlike the *rsaL* system where the expression of the repressor is dependent on LasR-AHL, *vioS* expression is not linked to CviR-AHL and the mechanism of *vioS* expression and regulation requires further investigation. In addition *vioS* is found in a separate genomic location from the *cviI* and *cviR* genes and does not have any direct effect on their transcription but impacts at an as yet unknown level on CviI/R QS target gene expression. Moreover, VioS appears to be sufficient and specific for CviR-AHL antagonism as it is not a general inhibitor of gene activation by other QS LuxR regulators in other bacteria, for example CepR-AHL from *Burkholderia*.

Sequence similarity searches with the predicted amino acid sequence of *C. violaceum* ATCC31532 VioS were undertaken to identify homologs of this protein in other bacteria. In our searches VioS homolog was identified only in the sequenced genomes of *C. violaceum* ATCC12472 strain and *P. ferrooxidans*. The exclusive presence of VioS in these two bacterial genera suggests that it may have specific functions in these bacterial species. In contrast, other QS repressors like RsaL and RsaM are present in multiple members of the proteobacteria (Venturi et al., 2011). Both *C. violaceum* and *P. ferrooxidans* produce the purple violacein pigment and it will be interesting to determine whether VioS also regulates pigment production in *P. ferrooxidans*. These two bacteria could share a similar niche(s) [*P. ferrooxidans* producing violacein has been isolated in a lake, {(Puranik, 2013) #736}] as well as profile and regulation of secondary metabolite production in order to survive in specific environmental conditions; this possibility is currently unknown. According to our experiments, the repressor function of VioS for violacein production is conserved in both *C. violaceum* ATCC31532 and the sequenced strain, *C. violaceum* ATCC12472 which however differ in the levels of violacein produced. We therefore decided to introduce the *vioS* gene of strain ATCC31532 *in trans* into the *C. violaceum* ATCC12472 wild type and this resulted in the transformation of the deep purple colony color to pale white color (Figure 1) indicative of violacein repression. Interestingly, *C. violaceum* ATCC12472 wild type has a gene homologous to *vioS* (CV_1055) and further experiments will be necessary to determine whether this genes codes for a functional protein or has lower expression levels than required to mediate its repressor effect in the presence of CviR-AHL. Interestingly, a very recent study has reported violacein production in the marine bacterium *Pseudomonas ulvae* and its regulation by AHL QS (Mireille Ayé et al., 2015). It would be interesting to determine whether VioS is present and regulates violacein production in this marine bacterium.

**Figure 5:**
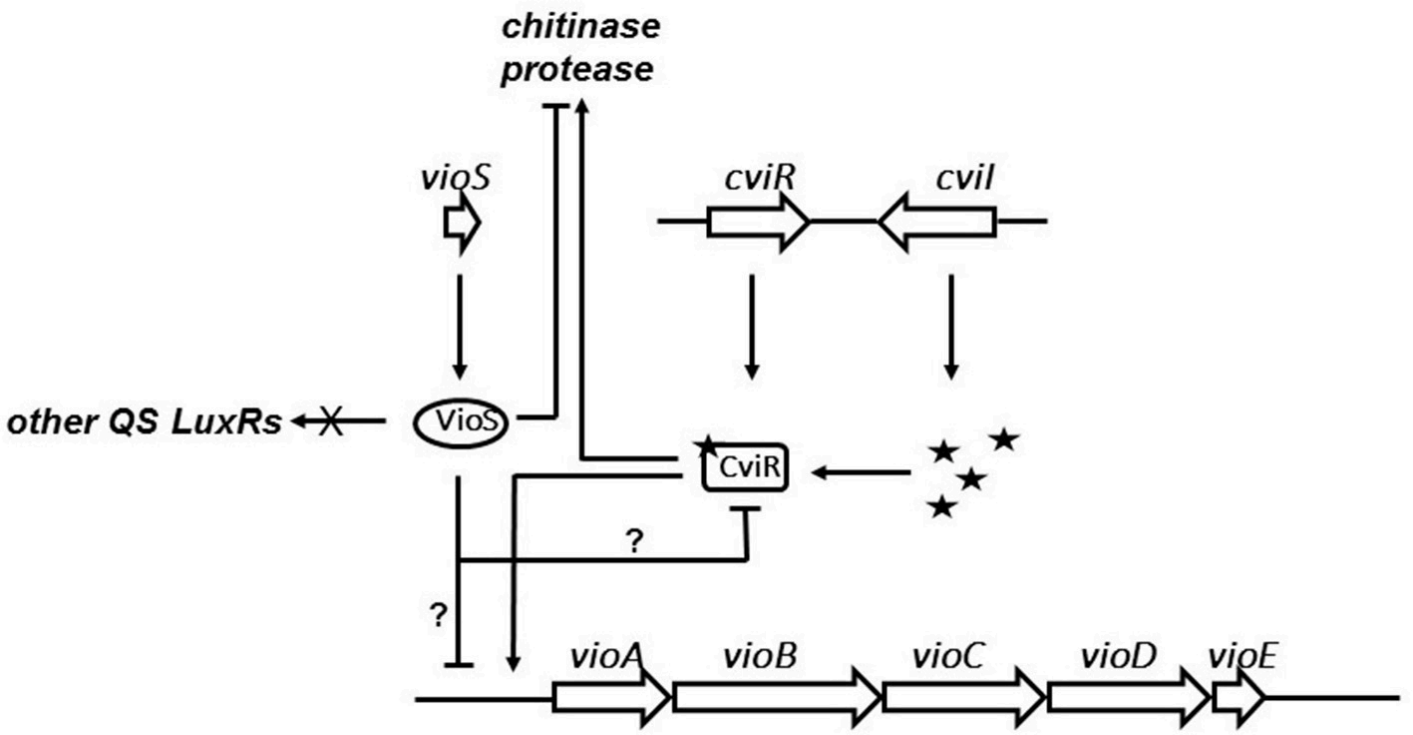
**Model for role of VioS in regulation of QS regulated phenotypes in *C. violaceum***. VioS negatively regulates the *vio* operon either directly or indirectly, which is positively regulated directly by the CviI/R QS system. VioS negatively regulates chitinase and protease production, which are positively regulated by the CviI/R system.

Our current understanding of VioS mediated regulation of violacein biosynthesis in *C. violaceum* is shown in the schematic model (Figure 5). Briefly, at high cell densities, the CviR protein binds AHLs to activate expression of *vioA* promoter in *C. violaceum* wild type. Expression of VioS under these conditions leads to repression of *vioA* promoter and consequently of violacein production and pale colonies of wild type *C. violaceum* ATCC31532. A *vioS* mutant is relieved from this repression at the *vioA* promoter leading to violacein production which is clearly visible as purple-colored colonies. Future studies need to address whether the effect of VioS on the *vioA* promoter is due to a transcriptional, post-transcriptional control or possibly via protein-protein interaction with the CviR-AHL complex. In addition from this study it is important to determine the levels of VioS required to antagonize CviR-AHL and the conditions that regulate *vioS* expression in *C. violaceum*.

## Author contributions

GD, MK, SC, and IB performed experimental work whereas MC, PW, SS, and VV drafted the manuscript. All authors were involved in designing, discussing, and interpreting the results of the experiments.

## Funding

We thank ICGEB for support.

### Conflict of interest statement

The authors declare that the research was conducted in the absence of any commercial or financial relationships that could be construed as a potential conflict of interest.
